# Dataset of the de novo assembly and annotation of the marbled crayfish and the noble crayfish hepatopancreas transcriptomes

**DOI:** 10.1186/s13104-022-06137-6

**Published:** 2022-08-22

**Authors:** Ljudevit Luka Boštjančić, Caterina Francesconi, Christelle Rutz, Lucien Hoffbeck, Laetitia Poidevin, Arnaud Kress, Japo Jussila, Jenny Makkonen, Barbara Feldmeyer, Miklós Bálint, Klaus Schwenk, Odile Lecompte, Kathrin Theissinger

**Affiliations:** 1 LOEWE Centre for Translational Biodiversity Genomics (LOEWE-TBG), Senckenberg Biodiversity and Climate Research Centre (SBiK-F), Georg-Voigt-Str. 14-16, 60325 Frankfurt am Main, Germany; 2 Institute for Environmental Sciences, University of Koblenz-Landau, Fortstrasse 7, 76829 Landau, Germany; 3 Department of Computer ScienceUMR 7357Centre de Recherche en Biomédecine de Strasbourg, ICube, University of Strasbourg, CNRS, Rue Eugène Boeckel 1, 67000 Strasbourg, France; 4 Department of Environmental and Biological Sciences, University of Eastern Finland, P.O. Box 1627, 70210 Kuopio, Finland; 5 Present Address: BioSafe - Biological Safety Solutions, Microkatu 1, 70210 Kuopio, Finland

**Keywords:** Freshwater crayfish, *Astacus astacus*, *Procambarus virginalis*, Crayfish plague, RNA sequencing

## Abstract

**Objectives:**

Crayfish plague disease, caused by the oomycete pathogen *Aphanomyces astaci* represents one of the greatest risks for the biodiversity of the freshwater crayfish. This data article covers the de novo transcriptome assembly and annotation data of the noble crayfish and the marbled crayfish challenged with *Ap. astaci*. Following the controlled infection experiment (Francesconi et al. in Front Ecol Evol, 2021, 10.3389/fevo.2021.647037), we conducted a differential gene expression analysis described in (Boštjančić et al. in BMC Genom, 2022, 10.1186/s12864-022-08571-z)

**Data description:**

In total, 25 noble crayfish and 30 marbled crayfish were selected. Hepatopancreas tissue was isolated, followed by RNA sequencing using the Illumina NovaSeq 6000 platform. Raw data was checked for quality with FastQC, adapter and quality trimming were conducted using Trimmomatic followed by de novo assembly with Trinity. Assembly quality was assessed with BUSCO, at 93.30% and 93.98% completeness for the noble crayfish and the marbled crayfish, respectively. Transcripts were annotated using the Dammit! pipeline and assigned to KEGG pathways. Respective transcriptome and raw datasets may be reused as the reference transcriptome assemblies for future expression studies.

## Objective

Freshwater crayfish are keystone species of freshwater habitats [1–3]. One of the major contributors to the loss of the European freshwater crayfish biodiversity is the introduction of highly competitive North American invasive crayfish species, carriers of the devastating disease crayfish plague [4]. This disease is caused by the oomycete pathogen, *Aphanomyces astaci* [5]. The noble crayfish, an endangered emblematic species of European freshwaters is considered to be highly susceptible to the pathogen [6]. On the other hand, the marbled crayfish, parthenogenetic species of North American origin is a known carrier of this pathogen [7]. In the controlled infection experiment described in [1], the marbled crayfish has been shown to be highly resistant to two *A. astaci* strains of differing virulence, Haplogroup B strain (Hap B; high virulence) and Haplogroup A (Hap A; low virulence). Concurrently, in the same experimental setup the susceptibility of the noble crayfish, especially to the lethal Hap B strain was confirmed. During the experiment, individuals of both species were sampled at: 3 dpi, 21 dpi for the analysis of the gene expression patterns in the infected individuals. Results of this study are presented in [2].

Here, we report a large collection of RNA sequencing data (55 samples) from the hepatopancreas of the noble crayfish and the marbled crayfish, and their de novo assembled and annotated transcriptomes. This data can provide insight into the biology of these two species and will allow for future comparative transcriptomic analysis. The datasets presented here can also serve as the reference transcriptomes for the future transcriptomic studies in the marbled crayfish and the noble crayfish and development of gene specific primers and expression assays. The dataset from the noble crayfish and marbled crayfish infected with *A. astaci* might be interesting to molecular Biologists, immunologists, bioinformaticians, evolutionary biologists and others interested in the innate immunity of the freshwater crayfish.

## Data description

### Data description

The data reported here represent an RNA sequencing dataset from *A. astaci* infected noble crayfish and marbled crayfish individuals [1]. Each sample represents a biological replicate, originating from a different individual. A total of 2430.7 million and 3098.2 million 2 × 150 bp paired-end reads (read depth: 36.8 M−68.9 M, mean: 48.59 M) were generated from the hepatopancreas of the noble crayfish and the marbled crayfish, respectively [8]. After processing of low-quality reads, a total of 2227.6 million (91.64% of the initial raw reads) and 2926.8 million (94.46% of the initial raw reads) high-quality sequences were retained for the noble crayfish and the marbled crayfish, respectively [9]. Raw read data are available at the NCBI database under SRA accession number: SRP318523 [8].

### Methodology

#### De novo transcriptome assembly

From the pooled Trinity de novo transcriptome assembly we obtained 670,741 transcripts for the noble crayfish (44,062 ORFs) and 11,333,173 (46,953 ORFs) transcripts for the marbled crayfish*.* In the post-assembly processing, after filtering fragmented transcripts 168,172 (44,062 ORFs) and 348,751 (46,953 ORFs) transcripts remained for the noble crayfish [10] and the marbled crayfish*,* respectively [11]. After redundancy reduction with CD-HIT-EST 109,608 genes and 254,336 genes remained for the noble crayfish and the marbled crayfish*,* respectively. BUSCO analysis of the final assembly revealed a high level of completeness for both assemblies, 93.30% for the noble crayfish and 93.98% for the marbled crayfish arthropoda_odb10 database of orthologs (n = 1013). Comparative analysis of the BUSCO scores among available freshwater crayfish transcriptomes placed the noble crayfish and the marbled crayfish transcriptome assemblies as the most complete freshwater crayfish transcriptome assemblies to date [12]. Length distribution of assembled transcripts varied from 401 to 32,629 in the noble crayfish and 401 to 32,816 in the marbled crayfish, with the highest number of transcripts falling in the category of 401–500 bp in length for both species [13]. The simple sequence repeats (SSRs) unit lengths ranged from 1 to 12, with 1 bp SSRs being the most abundant in the noble crayfish assembly and 2 bp SSRs in the marbled crayfish [13].

#### Transcriptome annotation

Gene model building using TransDecoder predicted 67,196 and 102,871 coding regions for the noble crayfish and the marbled crayfish*,* respectively. In total, 46,819 (69.7%) and 74,321 (72.2%) of the transcripts with predicted coding regions were annotated within the Dammit! pipeline when combining hits of all searches for the noble crayfish and the marbled crayfish*,* respectively [13]. Annotation features include putative nucleotide and protein matches in the OrthoDB, Pfam, UniRef90, Rfam and reference *Daphnia pulex* proteome.

As an additional approach for functional annotation, transcripts were mapped to the reference canonical KEGG database. For the noble crayfish, 13,336 transcripts were mapped across 426 pathways and for marbled crayfish 17,309 transcripts were mapped across 425 pathways [14]. Among the represented pathways, for both assemblies the highest number of transcripts was annotated to metabolic pathways, biosynthesis of secondary metabolites, microbial metabolism in diverse environments and pathways of neurodegeneration. Detailed methodological protocol is available [15].

## Limitations

Transcriptomic data allowed us to explore the gene expression landscape and identify key genes in the crayfish immunity. However, information about genomic locations and gene surroundings, which are highly influential on the gene expression profiles, are still not available. The quality of the transcriptomes could be improved by coupling these data with long-read sequencing data in future work to identify splice variants expressed during different experimental conditions. Furthermore, transcriptomic studies cannot address the real protein abundances, as changes in the gene expressions profiles are not always correlated to changes in the protein abundances.

## Data Availability

The data described in this Data note can be freely and openly accessed on the NCBI SRA, NCBI TSA and Figshare. Please see Table

**Table 1:** Overview of data files/data sets

Label	Name of data file/data set	File types (file extension)	Data repository and identifier (DOI or accession number)
*Data set 1*	*Astacus astacus and Procambarus virginalis Transcriptomes*	*FASTQ files (.fq)*	*NCBI SRA: *https://identifiers.org/insdc.sra:SRP318523 [8]
*Data set 2*	*Bostjancic_et_al_Data_set_2_Data_note*	*MS Excel file (.xlsx)*	*Figshare:* https://doi.org/10.6084/m9.figshare.15029001 [9]
*Data set 3*	*TSA: Astacus astacus, transcriptome shotgun assembly*	*FASTA files (.fasta)*	*NCBI TSA: *https://identifiers.org/nucleotide:GJEB00000000.1 [10]
*Data set 4*	*TSA: Procambarus virginalis, transcriptome shotgun assembly*	*FASTA files (.fasta)*	*NCBI TSA: *https://identifiers.org/nucleotide:GJEC00000000.1 [11]
*Data set 5*	*Bostjancic_et_al_Data_set_5_Data_note.tif*	*TIF file (.tif)*	*Figshare:* https://doi.org/10.6084/m9.figshare.15028644 [12]
*Data set 6*	*Bostjancic_et_al_Data_set_6_Data_note.tif*	*TIF file (.tif)*	*Figshare:* https://doi.org/10.6084/m9.figshare.15031779 [13]
*Data set 7*	*Bostjancic_et_al_Data_set_7_Data_note.tif*	*TIF file (.tif)*	*Figshare:* https://doi.org/10.6084/m9.figshare.15031773 [14]
*Data set 8*	*Bostjancic_et_al_Data_set_8_Data_note.pdf*	*PDF file (.pdf)*	*Figshare:* https://doi.org/10.6084/m9.figshare.15031776 [15]

1 and references [8–15] for details and links to the data. Overview of data files/data sets

## Abbreviations

Bp: Base pairs
BUSCO: Benchmarking sets of Universal Single-Copy Orthologs
Dpi: Days post infection
GEO: Gene Expression Omnibus
Hap A: Haplogroup A
Hap B: Haplogroup B
KEGG: Kyoto Encyclopedia of Genes an Genomes
NCBI: National Center for Biotechnology Information
ORFs: Open reading frames
OrthoDB: Ortholog database
Pfam: Protein family databse
Rfam: RNA family database
SSRs: Single sequence repeats
UniRef90: UniProt Reference Clusters

## References

[1] Francesconi C, Makkonen J, Schrimpf A, Jussila J, Kokko H, Theissinger K (2021). Controlled infection experiment with *Aphanomyces astaci* provides additional evidence for latent infections and resistance in freshwater crayfish. Front Ecol Evol..

[2] Boštjančić LL, Francesconi C, Rutz C, Hoffbeck L, Poidevin L, Kress A (2022). Host-pathogen coevolution drives innate immune response to *Aphanomyces astaci* infection in freshwater crayfish: transcriptomic evidence. BMC Genom..

[3] Reynolds J, Souty-Grosset C, Richardson A (2013). Ecological roles of crayfish in freshwater and terrestrial habitats. Freshw Crayfish.

[4] Holdich DM, Reynolds JD, Souty-Grosset C, Sibley PJ (2009). A review of the ever increasing threat to European crayfish from non-indigenous crayfish species. Knowl Manag Aquat Ecosyst.

[5] Alderman DJ (1996). Geographical spread of bacterial and fungal diseases of crustaceans. Rev Sci Tech l’OIE..

[6] Becking T, Mrugała A, Delaunay C, Svoboda J, Raimond M, Viljamaa-Dirks S (2015). Effect of experimental exposure to differently virulent *Aphanomyces astaci* strains on the immune response of the noble crayfish *Astacus astacus*. J Invertebr Pathol.

[7] Keller NS, Pfeiffer M, Roessink I, Schulz R, Schrimpf A (2014). First evidence of crayfish plague agent in populations of the marbled crayfish (*Procambarus fallax* forma virginalis). Knowl Manag Aquat Ecosyst.

[8] Boštjančić LL, Francesconi C, Rutz C, Hoffbeck L, Poidevin L, Kress A (2022). NCBI Sequence Read Archiv.

[9] Boštjančić LL, Francesconi C, Rutz C, Hoffbeck L, Poidevin L, Kress A (2022). Figshare.

[10] Boštjančić LL, Francesconi C, Rutz C, Hoffbeck L, Poidevin L, Kress A (2022). NCBI TSA:.

[11] Boštjančić LL, Francesconi C, Rutz C, Hoffbeck L, Poidevin L, Kress A (2022). NCBI TSA:.

[12] Boštjančić LL, Francesconi C, Rutz C, Hoffbeck L, Poidevin L, Kress A (2022). Figshare.

[13] Boštjančić LL, Francesconi C, Rutz C, Hoffbeck L, Poidevin L, Kress A (2022). Figshare.

[14] Boštjančić LL, Francesconi C, Rutz C, Hoffbeck L, Poidevin L, Kress A (2022). Figshare.

[15] Boštjančić LL, Francesconi C, Rutz C, Hoffbeck L, Poidevin L, Kress A (2022). Figshare.

